# Precomputed Radiative Heat Transport for Efficient Thermal Simulation

**DOI:** 10.1111/cgf.14957

**Published:** 2023-11-05

**Authors:** C. Freude, D. Hahn, F. Rist, L. Lipp, M. Wimmer

**Affiliations:** ^1^ TU Wien, Institute of Visual Computing and Human‐Centered Technology Austria; ^2^ King Abdullah University of Science and Technology, Visual Computing Center Kingdom of Saudi Arabia

**Keywords:** CCS Concepts, • ***Computing methodologies*** → ***Ray tracing; Physical simulation***, • ***Applied computing*** → *Physics; Computer‐aided design*

## Abstract

Architectural design and urban planning are complex design tasks. Predicting the thermal impact of design choices at interactive rates enhances the ability of designers to improve energy efficiency and avoid problematic heat islands while maintaining design quality. We show how to use and adapt methods from computer graphics to efficiently simulate heat transfer via thermal radiation, thereby improving user guidance in the early design phase of large‐scale construction projects and helping to increase energy efficiency and outdoor comfort. Our method combines a hardware‐accelerated photon tracing approach with a carefully selected finite element discretization, inspired by precomputed radiance transfer. This combination allows us to precompute a radiative transport operator, which we then use to rapidly solve either steady‐state or transient heat transport throughout the entire scene. Our formulation integrates time‐dependent solar irradiation data without requiring changes in the transport operator, allowing us to quickly analyze many different scenarios such as common weather patterns, monthly or yearly averages, or transient simulations spanning multiple days or weeks. We show how our approach can be used for interactive design workflows such as city planning via fast feedback in the early design phase.

## 1. Introduction

Urban planning and architectural design are challenging design tasks, since complex physical inter‐dependencies can result in unforeseen consequences. Modern construction projects often comprise complex structures and highly reflective materials, which give rise to complex radiative transport effects that can lead to a concentration of heat in certain areas of the design. A popular example is the 20 Fenchurch Street skyscraper in London, which originally exhibited the so‐called *Solar glare problem*. Its highly reflective and concave glass facade focused sunlight on a small area on the streets below, which subsequently raised the temperature there to problematic levels. This issue had to be fixed by retrofitting the skyscraper with permanent awnings. Simulation can help to predict such problems before construction and show how heat radiation can affect a design. Such simulations need to be efficient and interactive to effectively guide the designer and help avoiding problems like heat islands and improving energy efficiency of designs. Furthermore, when structures are designed carefully, reflecting materials can also be used to guide radiation into areas which would otherwise receive less or not enough light or heat. In general, being able to efficiently simulate these phenomena can help making city designs more energy efficient by distributing sunlight more intelligently, as well as reducing the need for additional heating or cooling.

**Figure 1:**
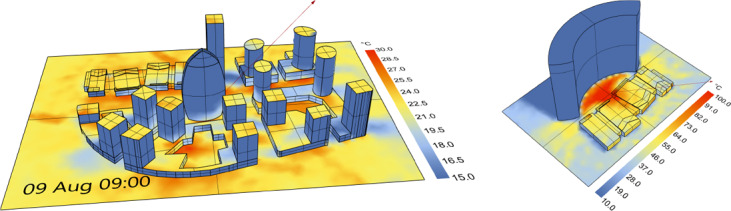
Our approach can efficiently compute transient (left) or steady‐state (right) radiative heat exchange in scenes with complex geometry and arbitrary transport, i.e., non‐diffuse or glossy materials. We incorporate measured sky radiation data and efficiently cache the radiative transport, allowing for fast computation of arbitrary day/time/weather configurations, thereby enabling rapid design iteration and prototyping. Here, we show the surface temperature distribution over multiple city blocks for a particular time step during a transient thermal simulation (left). A steady‐state solution (right) reveals a potentially problematic “hot spot” due to the highly specular reflective and concave facade design.

Due to the complexity of radiative heat transport, many common tools in practice only account for direct and diffuse radiation, while ignoring indirect radiation and glossy reflections. Conversely, modern rendering methods excel in solving indirect illumination and general reflective surfaces, but typically only deal with the visible part of the electromagnetic spectrum instead of thermal radiation. These rendering approaches are therefore ineffective for the thermal analysis of designs currently, but point the way to solve indirect thermal transport including non‐diffuse materials. Furthermore, existing tools are often complicated to use and too slow for interactive feedback. Consequently, we want to provide an efficient thermal simulation solution that can aid designers in early planning stages by providing an interactive simulation framework including indirect irradiation and glossy reflections. We aim at giving intuitive guidance on how thermal radiation affects heat distribution during the development of early designs. To this end, we build upon methods from computer graphics and rendering and show how they can be extended and adapted for the efficient simulation of thermal radiation.

Physically‐based rendering is a well‐established branch of computer graphics, simulating how visible light scatters throughout a 3D scene. While rendering methods commonly account for emitted and reflected radiation, they generally ignore the *absorbed* radiation. Furthermore, emission (i.e., a light source) is modeled as a fixed scene parameter, independent of the computed solution. While these assumptions are sufficient to produce accurate images of a scene, some applications, e.g., in engineering and architecture, also require the computation of *temperatures* on surfaces. Temperature changes dynamically due to differences in absorbed and emitted radiation, while emission is itself driven by surface temperature. *Thermal radiation simulation* is therefore the process of accurately computing these effects, together with the resulting temperature distribution. As all objects with a temperature above absolute zero emit radiation, an accurate simulation of thermal radiation requires the computation of temperature distributions across the whole scene. So, in the thermal context, we not only need to deal with the global nature of light transport as in classical physically‐based rendering, but also with the fact that every part of the scene potentially affects the temperature and subsequently the emitted radiation of every other part of the scene. This inherently global and non‐linear interdependence makes the efficient simulation of thermal radiation especially challenging. Furthermore, we need to consider the total radiative power, integrated over the entire spectrum, instead of narrow bands of visible colors.

In this paper, we present a method for efficiently simulating the heat transfer between surfaces through thermal radiation. We designed our approach towards the requirements of the early‐phase architectural design process, in which materials and geometry are only roughly defined, and fast simulation feedback is required to aid the iterative design workflow. Furthermore, simulation of temperature changes over multiple days or months is necessary for architectural design evaluations, requiring fast and highly efficient computational approaches. Our method supports indirect radiative transport, as well as non‐diffuse materials exhibiting both diffuse and glossy reflections. Our prototype currently implements a mixture of diffuse and ideally specular materials. By pre‐computing the radiative transport, we can quickly compute surface temperatures across the whole scene geometry, and support efficient calculation of transient and steady‐state solutions. Furthermore, we incorporate solar irradiation from commonly used data sources with static geometry, which integrates into our pre‐computation scheme. In this way, we can reuse our transport operator to simulate time‐dependent solar irradiation efficiently. Therefore, we can quickly compute temperature distributions throughout the scene, for a large set of different sun and sky conditions at specific points in time, or for static averages of irradiation data. Our approach is inspired by, and a combination of, the *finite element method, photon tracing* and *precomputed radiance transfer (PRT)*, extending these ideas for efficient radiative heat transport, which requires specific, novel adaptations. According to a finite element discretization, we compute and store the temperature distribution on a per‐vertex basis of the surface geometry. As surface temperature and emitted energy are related non‐linearly by a fourth‐order term, we specifically choose a vertex‐associated piecewise‐constant approximation, which greatly simplifies the calculation of this term. The precomputed radiative transport is represented by a matrix that encodes the potential energy exchange between vertices and their associated triangles and is computed via *photon tracing*. We use modern graphics processing unit (GPU) hardware ray tracing to accelerate this photon tracing step. In summary, our contributions are:

1. A framework that combines *finite elements* and *photon tracing* to pre‐compute radiative transport of direct and indirect radiation incorporating diffuse and glossy scattering.
2. A specialized geometry assembly for the sky which, in conjunction with our pre‐computation scheme, enables fast sun and sky irradiation updates.
3. An efficient non‐linear thermal solver, leveraging piecewise‐constant basis functions to avoid coupled non‐linearities due to *T* ^4^ terms.
4. An integration of our framework into the popular computer‐aided design (CAD) tools *Rhino* and *Grasshopper* [Rhi].

We summarize the theoretical background in §3 and describe details of our method, implementation, and integration with *Rhino* in §4 and §5, before showing an evaluation of our method against *Elmer FEM* [CSC23] and examples for urban planning in §6.

## 2. Related Work

### 2.1. Radiosity, FEM, and Precomputed Radiance Transfer

Common approaches for solving radiative heat transfer include finite element method (FEM)‐based methods [Wie66, LB13], most notably *Radiosity*. In computer graphics, this method was first applied by Goral et al. [GTGB84] to compute light transport in complex scenes. The idea of Radiosity is to discretize the geometry into surface patches, and compute so called view (or form) factors, which are coefficients for the amount of direct radiation exchanged between pairs of patches. These form factors are subsequently used to build a linear system (and corresponding matrix) which is solved to compute the radiosity for each patch. This linear system can be solved by inversion, but more commonly is approximated iteratively [CCWG88]. The original form of radiosity only supports diffuse surfaces, while more advanced extensions also handle nondiffuse transport [ICG86, SAWG91, BP93]. However, using more complex basis functions or support for non‐diffuse transport increases the computational overhead. Various optimizations have been developed that improve the computational complexity and memory requirements, such as adaptive meshing [LTG92], hierarchical [HS92] and adaptive subdivision schemes [DB95], progressive refinement [NFKP95], efficient support for dynamic scenes [BBS95,FYT95,ÁDE96, BMA03, CzSG08], and accelerated view factor computation on the GPU [TYSK06,KGP*15]. While the latter two build upon rasterization‐based visibility testing on the GPU to compute direct view (or form) factors between two elements, we compute complete multi‐bounce transport between any two elements via *photon tracing*, inspired by the *Gebhart method* [Geb61]. Dachsbacher et al. [DSDD07] proposed an intriguing and fundamentally new radiosity approach based on so‐called antiradiance, which alleviates the need for explicit visibility calculations. Even though it can be accelerated using the GPU, the use of antiradiance is less suited for ray‐tracing‐based approaches. Another fundamentally different approach to radiosity was presented by Hadadan at al. [HCZ21], which uses a neural network to solve the rendering equation. It provides high‐quality and view‐independent solutions; however, training can take up to several hours. In contrast, our approach uses accelerated RTX ray tracing to compute thermal transport for quick iterative workflows.

In the context of thermal building analysis, Dionisio et al. [DADG96] presented a method to compute the solar influence on buildings based on a two‐pass radiosity approach [SP89]. In the first pass, a radiosity solution is computed, and in the second, view‐dependent pass, the final image is rendered using ray tracing. Our approach computes a *view‐independent* solution, similar to the concept of *extended view factors* [SP89,SAWG91] to support specular transport. However, we compute these factors by GPU photon tracing using recent RTX accelerated ray tracing.

Our approach is conceptually similar to shooting [CCWG88] or stochastic radiosity [NFKP95], in that, we use stochastic photon tracing starting from the emitting surfaces. Radiative heat transport, however, requires specific solutions and adaptations. While rendering methods solve for reflected radiance, here we consider temperature, which is affected by absorbed and emitted (rather than reflected) radiation. This difference has two key consequences: (1) temperature is a scalar field (i.e., it does not depend on the viewing direction), so extensions of radiosity to directional radiance fields do not apply here. Therefore, (2) reflected (directional) radiation cannot be represented by the temperature field, which means that we cannot find an infinite‐bounce solution including glossy reflections by inverting a transport operator containing pair‐wise form factors resulting from direct visibility. Our solution to this problem is to instead include indirect reflections in the transport operator itself. Heat transport is not a linear problem like lighting, due to the highly non‐linear relationship between temperature and emitted power. Furthermore, we require a global surface temperature field, and cannot afford to use iterative schemes by recomputing the radiosity solutions (or re‐trace photons) in every iteration of the nonlinear thermal system solver. For our proof‐of‐concept implementation, we did not use any adaptive meshing, or incremental update or computation schemes, but consider such optimizations as future work.

**Figure 2:**
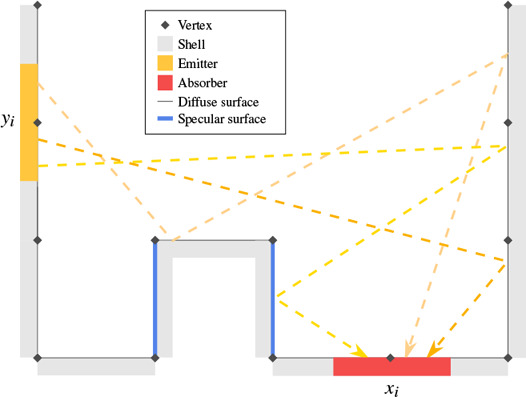
Here we illustrate the construction of our transport operator, which includes radiance reflected over multiple full paths from the area around an emitting vertex y_i_ to the area around an absorbing vertex x_i_.

Instead, we focus on developing a thermal transport operator that encodes transport of all possible paths between patches (see Fig. 2), including non‐diffuse reflections, for which we do not need to solve the transport equation iteratively, but can pre‐compute it just once and reuse it for each thermal time step as long as the scene geometry does not change. Therefore, our approach is similar to the idea of precomputed radiance transfer (PRT) [SKS02, LSSS04, Leh07], since it also pre‐computes and caches the transport operator. However, as stated before, radiative heat transport is highly non‐linear, in contrast to linear light transport. Furthermore, in the case of thermal radiation, *all* surfaces are emitters, whereas PRT methods commonly consider only distant environment maps as light sources. Additionally, since we compute a global (view‐independent) temperature distribution on the scene surfaces, using directional basis functions does not apply in our context. Even if such a directional formulation could be applied, it would vastly increase the memory and computation overhead. Instead, our thermal transport operator includes diffuse and glossy reflections between all surfaces without the need for directional data structures, as illustrated in Figure 2.

### 2.2. Monte Carlo Ray tracing

Nowadays, Monte Carlo integration and ray tracing are the defacto standard in computer graphics for the generation of photorealistic images. One of the most common approaches is *path tracing* [Kaj86], which supports arbitrary transport and requires no surface discretization. It is commonly used to compute view‐dependent solutions, i.e., to render an image. Random paths starting from the camera are traced through the scene to estimate the amount of light reaching the image sensor. Complementary approaches, where the random paths start from the light sources, include *light tracing* [Arv86] and *photon mapping* [Jen96]. The latter first uses a *photon tracing* pass to store incident photons in a *photon map*, and in a second pass uses density estimation based on nearby photons to reconstruct the incident illumination during ray tracing of the final image.

While such approaches are predominantly used to compute photo‐realistic images in the visible spectrum, they have also been applied for the simulation of thermal radiation [How98] in the context of semiconductors [MK00], gases [KIB05], fluids [Sem10], and melting of ice [FM07]. For the latter two, *photon mapping* was used. In the urban context, ray tracing has been applied in combination with FEM by various approaches. However, they either only consider diffuse scattering [KV07,YL13] or do not cache the transport operator for efficient transient simulations [OWB*16].

For our approach, we also use *photon tracing*, but since we are only interested in a non‐directional temperature distribution on surfaces, we do not need a photon map for density estimation and therefore accumulate the absorbed photon energy directly for each vertex on the surface.

### 2.3. Building Energy Simulation

Efficiently modeling and simulating the energy transport for complex buildings is a challenging task. For this purpose, complex software frameworks have been developed such as *EnergyPlus* [CPLW00] or *Sustain* [GPH*13]. Another popular collection of tools are the *Ladybug Tools* [Lad, MGNR17], a suite of plugins for *Grasshopper* [Gra] and *Rhino3D* [Rhi] that build on *EnergyPlus* [CPLW00] and *Radiance* [WS98]. Simulation of radiation often only considers direct solar radiation [JGP12, MPLFC13, AYG*20] and employs simplified models and calculation, e.g., polygon clipping or pixel counting [HD17,dOM17,dRO*19]. Only a few works use GPU acceleration [JR14b, JR14a, JR15, JR17] or consider indirect radiation reflected by surrounding buildings or the environment and simulate thermal radiation [AGNA*20, CSF*22].

Those most recent approaches deal with the simulation of thermography imaging [AGNA*20] or computation of view‐dependent solutions or statistical aggregates [CSF*22]. In contrast, we focus on the efficient computation of view‐independent results, i.e., the temperature distribution over all surfaces due to, e.g., direct and indirect solar radiation. This can facilitate rapid iterations in the early design phase, in which quick feedback processes are important [JR19], making efficient computation essential. Despite close similarities between physically‐based rendering and simulation of radiative heat exchange [Dom08], the application of advanced accelerated rendering techniques for thermal simulation is rare.

Our approach combines the *finite element method* with GPU‐accelerated *photon tracing* to simulate direct, as well as indirect, thermal radiation for diffuse and specular reflecting surfaces. Furthermore, our approach supports fast computation of transient simulations by caching the radiation transport.

## 3. Theory of Radiative Heat Transport

In this work, we focus on the simulation of *radiative* heat transport. This section summarizes the relevant theory of grey‐body emission, transport including indirect reflections, and temperature changes due to absorbed radiation.

Matter at any temperature above absolute zero (0° K) emits electromagnetic radiation according to the Stefan–Boltzmann law [Ste79, Bol84]. Kirchhoff's law [Kir60] states that the absorptivity must equal the emissivity ε to allow thermal equilibrium. Ideal *black bodies*, which absorb all incident radiation have emissivity ε = 1, and emit according to Planck's black‐body spectrum [PM14]. Conversely, objects with ε < 1 are called *gray bodies*.

Specifically, given the local temperature *T* at a point *x* on the surface of a solid *grey body*, the *radiant exitance* (or radiant thermal flux per area), integrated over the entire spectrum, is

(1)

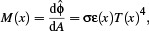




where 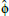 is the emitted *radiant flux*, σ is the Stefan–Boltzmann constant and ε is the material's emissivity. Assuming diffuse emission, the exitant radiance *L_e_* is constant over all exitant directions and

(2)

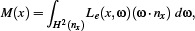




where *H* ^2^(*n_x_*) is the hemisphere oriented along the normal *n_x_* of the surface at location *x*. Consequently, we find

(3)

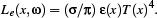




The rendering equation [Kaj86] relates incident radiance *L_i_* arriving at *x* from direction ω_*i*_ to radiance leaving *y* in direction ω_*o*_ = –ω_*i*_ by *L_i_* (*x*,ω_*i*_) = *L*(*y*,ω_*o*_) if *y* is visible from *x* in direction ω_*i*._ The radiance leaving *y* is

(4)

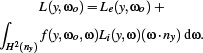




Here, *f*(*y*,ω_*o*_,ω) is the material's surface bidirectional reflectance distribution function (BRDF) [Kaj86, PJH16]. One challenge is to compute the incident radiance *L_i_* in a potentially complex multi‐body environment with glossy reflective materials (see Section 4.2). Additionally, in contrast to common rendering, the emissive term *L_e_* in the context of thermal radiation also depends on the resulting temperature. Gathering incident radiance, *L_i_*, arriving at *x* from all directions yields the *irradiance* (i.e., received thermal flux per area)

(5)

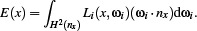




The amount of absorbed *irradiance* (absorbed flux density) is then

(6)

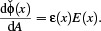




Combining absorption, Eq. (6), and emission, Eq. (1), yields the net flux density, dφ/d*A*, at a point *x*, i.e. the difference between absorbed and emitted power per area:

(7)

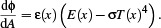




Intuitively, this difference determines if an object gains or loses energy and subsequently heats up or cools down.

We now relate this net radiative flux to the physical material properties to determine the resulting rate of temperature change. Throughout this work, we assume a relatively thin object with small thickness *h*, such that the temperature variation orthogonal to the surface is negligible. We also assume that bulk material properties do not change in the direction orthogonal to the surface. Consider a small surface patch d*A*, and the corresponding shell volume *h* d*A*: the thermal energy density due to a given temperature is d*Q*/d*A* = *Tc_p_* ρ*h*, where *c_p_* is the material's specific heat capacity and ρ its mass density. Noting that flux density (dφ/d*A*) is the time derivative of energy density (d*Q*/d*A*), we find dφ/d*A* = d(d*Q*/d*A*)/d*t* = (d*T*/d*t*)*c_p_* ρ*h*. Substituting from Eq. (7), we find the change of temperature over time:

(8)

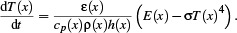




Discretizing this temperature change on the surface (as well as in time) enables us to simulate surface temperatures over time (transient simulation), or determine the stable temperature distribution (steady state). In the next section, we explain how we represent the surfaces and compute the *irradiance E* for use in an efficient thermal simulation.

## 4. Method

The goal of our approach is to efficiently compute surface temperatures (steady state or transient) for a given input scene defined by triangle meshes and associated material properties including initial temperatures. We compute temperatures and cache the radiation transport (operator) based on a finite element discretization using the mesh vertices. The radiation transport (operator) is precomputed via *photon tracing*.

Before we go into more detail about our method, we first summarize our assumptions. As outlined in the previous section, we assume that objects can be modeled with thin shells representing their radiating surfaces. While we work with thin triangles geometrically, the thermal mass resulting from the structural thickness is taken into account, and can in theory be set to represent the whole volume of the building if required. Furthermore, due to heating and cooling systems, the interior of buildings can be loosely regarded as being at a constant temperature (e.g. 22°C), and its internal structure does not need to be modeled in detail in the context of the simulation of a whole city block. The influence of this assumed internal temperature depends on the building's insulation. For now, we have assumed ideally insulated facades, but our method could be extended to include this thermal coupling, or be combined with an interior thermal model beyond the scope of our current work. For our prototype implementation, we assume that the material properties are uniform across wavelengths, which is not true in general. However, for temperature simulation, we are primarily dealing with quantities integrated over the full spectrum, where a gray‐body model captures absorbed and emitted total power. As far as reflections are concerned, our model assumes wavelength‐independent geometric optics (common in rendering), which is sufficient for our intended application of early‐design planning, where details of the used materials are not fully specified yet. Technically, our approach could be extended to simulate multiple wavelength bands independently.

Following the theory presented in the previous section, we describe our finite element discretization in Section 4.1, which results in a convenient diagonalization of the non‐linear *T* ^4^‐term. This discretization gives rise to a transport operator *T*, which remains constant as solar irradiation changes over time. Afterward, we show how to pre‐compute this operator, employing hardware‐accelerated *photon tracing*, in Section 4.2. And finally, we describe how to incorporate radiation from a sky model, in Section 4.3.

### 4.1. Finite Element Discretization

In order to simplify calculations involving the fourth‐order term *T*(*x*)^4^ in Eq. (8), we choose a vertex‐centric, piecewise‐constant finite element approximation scheme. Specifically, we describe a volumetric shell in 3D by a collection of triangular surface elements. We then associate every vertex *x_i_* with one third of each of its adjacent triangles. More formally, we consider indicator functions *I_i_* (*x*), where *I_i_* (*x*) = 1 for all *x* whose barycentrically nearest vertex is *x_i_* (and zero otherwise), i.e., points are associated with the vertex with the highest barycentric coordinate within each triangle. Consequently, the functions *I_i_* have compact support and form a partition of unity. The vertex‐associated area at *x_i_* is then


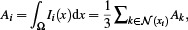




where Ω refers to the entire simulation domain, *𝒩*(*x_i_*) denotes the triangles adjacent to vertex *x_i_*, and *A_k_* refers to the area of each triangle. Using these indicators as interpolation basis functions, the approximate surface temperature field is

(9)

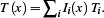




Applying this discretization scheme to Eq. (8), as detailed in our supplementary document, yields

(10)

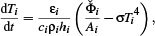




where {ε_*i*_,*c*_*i*_,ρ_*i*_,*h*_*i*_} are (piece‐wise constant) material properties near *x_i_* (emissivity, specific heat capacity, mass density, and shell thickness, respectively). Note that (*I_i_* (*x*)*T_i_*)^4^ = *T*_*i*_^4^ for *I_i_* (*x*) = 1 and at any point *x* exactly one of the terms in Eq. (9) is nonzero. This approach has the advantage that we do not have to linearize and solve a more complex interpolation term of the form (Σ_*i*_ *T*_*i*_ *I*_*i*_(*x*))^4^ *I*_*j*_(*x*) as would be necessary in case of linear interpolation. Furthermore, the vertex‐associated incident radiant flux per area 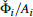, required to evaluate Eq. (10), follows from integrating irradiance around vertex *i*:

(11)

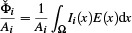



(12)

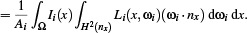




Finally, using a path integral formulation [Vea98], we can write the incident radiance *L_i_* due to radiation emitted from all vertices *j*:

(13)

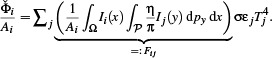




**Figure 3:**
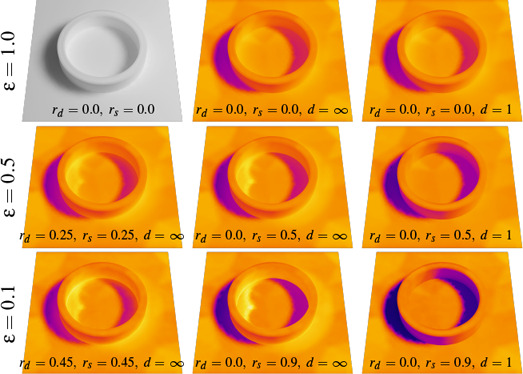
This example illustrates how our transport operator encapsulates full paths including specular reflections, showing varying emissivity ε, material coefficients (diffuse r_d_, specular r_s_), and path depth d. The top‐left image shows a result computed with Elmer FEM (ideally‐diffuse reference), all others were computed in our framework. From top to bottom, the emissivity ε decreases from 1 (black body) to 0.1 (highly reflective grey body). In the left and middle columns, the transport operator includes full paths with arbitrary length (d = ∞). The resulting heat island (caustic) inside of, and specular reflections around the ring are clearly visible. The right column compares these results to including direct radiation only (d = 1) in the transport operator; note how specularly reflected radiation is lost when ε < 1.

Here, we define the incidence matrix **F** = (*F_i j_*), where each entry encapsulates the radiative transport from point *y* (associated with vertex *j*) to point *x* (near vertex *i*), along *all* paths *p_y_ ∈ 𝒫* connecting *x* and *y* (including via indirect reflections) with path throughput η, according to Pharr et al. [PJH16]. Note that the emitted radiance due to temperature *T_j_* introduces 1/π, Eq. (3), which is accounted for during *photon tracing* in practice. In this formulation, *F_i j_* can be interpreted as a generalized view factor or *Gebhart factor* compared to standard, diffuse‐only, radiosity methods. We solve this complex transport problem by *photon tracing*, as detailed in Section 4.2. Note that, in contrast to traditional view factors, our solution also includes indirect, and possibly specular, reflections in the transport simulation. This is illustrated in Figure 2 and 3.

Denoting the vector of all per‐vertex temperatures as **T** = (*T_i_*), and similarly, the vector of (element‐wise) fourth powers of temperature as **T** ^4^ = (*T*_*i*_^4^), we can concisely write the time evolution of the unknown temperature variables as

(14)

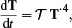




where *𝒯* is the *radiative transport operator*. It is composed of the incidence **F**, as well as the emissive term and material constants from Eq. (10) as follows:

(15)

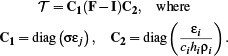




Here **I** is the identity matrix, accounting for energy lost due to emission at each vertex; both **C**_1_ and **C**_2_ are diagonal matrices, scaling the rows and columns of (**F** – **I**) respectively, according to the material constants and shell thickness associated with each vertex.

In summary, when applied to the vector of fourth powers of current temperatures **T** ^·4^, the transport operator describes the resulting rate of temperature change throughout the scene. Please note that in our approach, multi‐bounce indirect transport is already encoded in the transport operator, Eq. (13), which allows us to simulate specularly reflective materials efficiently, as demonstrated in Figure 7. While the transport operator scales quadratically in the number of degrees of freedom, fast hierarchical approximation methods [KRR02, GH03] can make the complexity manageable; we have not needed to do so for our results. Conversely, if the data structure were to include directional degrees of freedom, the system size would quickly grow with directional resolution. Our approach avoids this issue.

So far, we have constructed a piece‐wise constant finite element formulation, which forms the basis of our method. We now turn towards pre‐computing the transport operator, Eq. (13) and (15), before solving transient as well as steady‐state heat flow problems in Section 5.

### 4.2. Pre‐computing the transport operator

Monte Carlo integration uses random sampling to integrate complex functions. In general, the integral *I* of an arbitrary multidimensional function *f* is approximated as

(16)

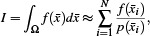




where Ω is a subset of *R^m^* and *N* is the number of random samples *x̄_i_* drawn from distribution *p* used to sample *f* and approximate the integral. In this section, we describe how we construct the radiative transport operator, *𝒯*, applying Monte Carlo (MC) integration to Eq. (5) in a *photon tracing* framework.

Intuitively, the transport operator in Eq. (14) can be interpreted as follows: each *column* of *𝒯* describes how energy emitted around the corresponding vertex is distributed throughout the scene. Conversely, each row describes how energy from around the scene is affecting the temperature at the corresponding vertex. Consequently, we build the transport operator by tracing *photons*, i.e., packets of unit energy flux (power), through the scene as follows: We emit a fixed number of photons around each vertex (distributed uniformly over the adjacent area) and in random directions (distributed cos‐weighted over the hemisphere), since thermal radiation is generally diffuse. Each photon represents an equal fraction of the power emitted around the corresponding vertex. We then trace the path of each photon through the scene. If a photon hits another surface, we determine via *Russian Roulette* [AK90] whether it is absorbed or reflected, where the probability of absorption is ε (i.e., equal to emissivity) and conversely, the probability of reflection is 1 – ε. In the latter case, the reflection direction is chosen randomly according to the BRDF of the material.

Finally, if the photon is absorbed, we accumulate the photon's contribution at the vertex corresponding to the hit location. In this way, each complete photon path represents a fractional energy flux from the emitting to the absorbing vertex, under the assumption of unit temperature at the emitter. Summation of all such contributions at the absorbing vertices produces the incidence matrix **F** = (*F_i j_*) as in Eq. (13).

### 4.3. Solar Irradiation

The simulation of thermal radiation in the context of architectural design requires to account for radiation from the sun and sky, changing over time. The corresponding radiation can be computed for example via *Radiance GENDAYMTX*, which returns radiance values for discrete patches of an approximation (subdivision) of the sky dome.

In principle, we could implicitly query this representation during ray or photon tracing, or convert it to an environment map, as commonly used in classical rendering for environment lighting. However, we choose to integrate solar irradiation via a fixed geometry representing the sky dome. This geometry enables a unified representation of all sources of thermal radiation, i.e., buildings as well as the sky and sun environment, and does not require our prototype implementation to deal with different special cases during computation. Furthermore, it leverages our pre‐computed transport operator and enables us to change the solar irradiation, for example for different time or day, by simply (re‐)assigning radiation values without the need for expensive re‐computation of the radiative transport.

**Figure 4:**
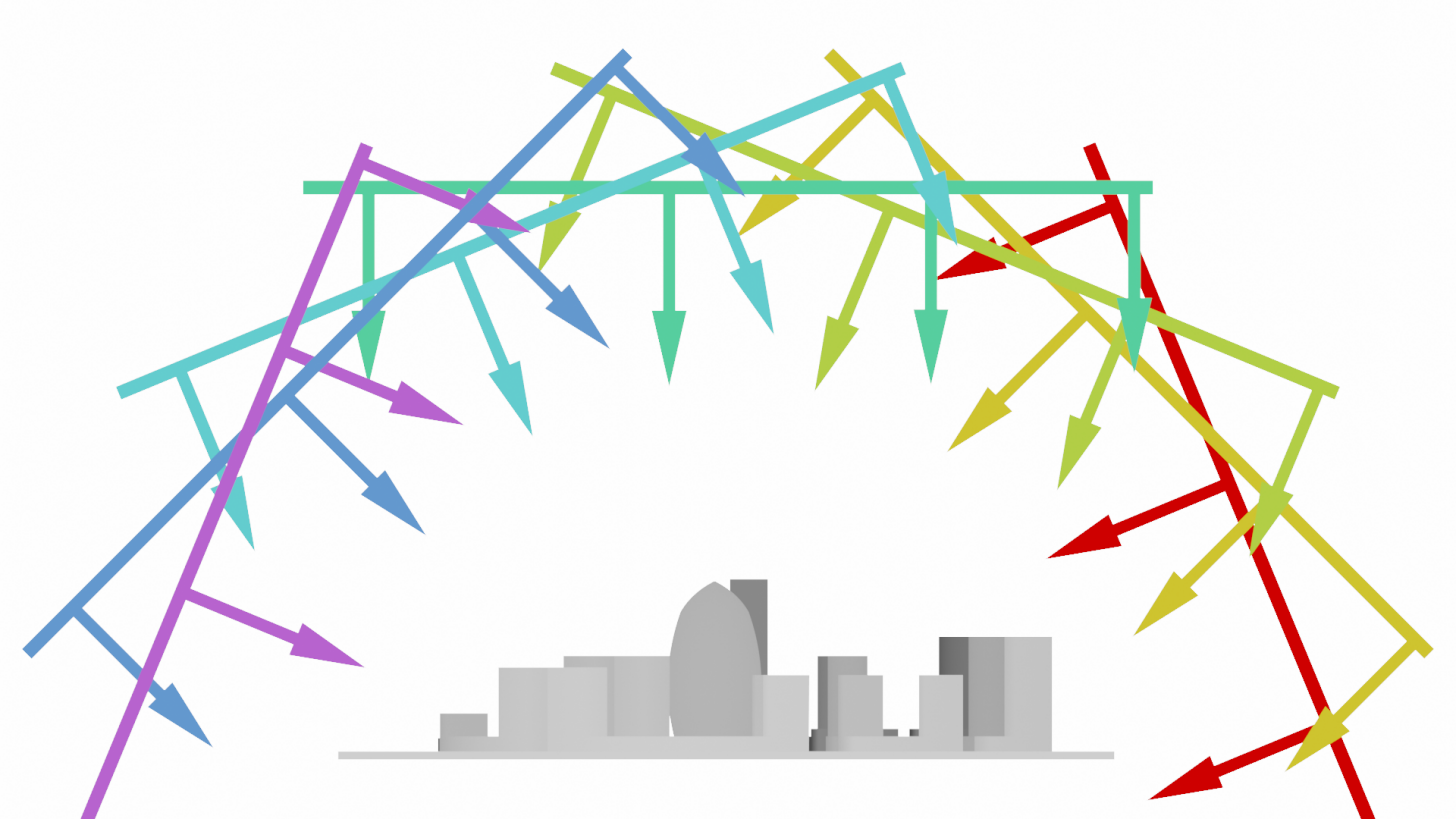
Illustration of the geometric approach used to represent the sky dome and its radiation. Each patch of the dome emits parallel rays but is excluded from intersection testing; each patch also covers the whole scene.

We construct this fixed geometry, illustrated in Figure 4, as follows: Given a subdivision of the sky dome into patches with corresponding radiance values, e.g., from *Radiance GENDAYMTX*, we generate geometry quads corresponding to each sky patch. Each quad represents a distant parallel emitter placed on a hemisphere around the scene in the direction of the corresponding sky patch. In order for the photons emitted from this quad geometry to cover the whole scene, the quads are scaled to the size of the scene bounding sphere, and their normals are oriented towards the scene center, with photon emission set to parallel along the normal. Since this geometry is included in the pre‐computation of the transport operator, any sky configuration can be efficiently simulated by simply (re‐)assigning temperature values corresponding to the radiosity values of the sky patches. For the conversion from a perpatch radiosity value *J_e_*, representing full‐spectrum incident radiation, to an effective temperature (in Kelvin), we assume black‐body radiation and compute the temperature according to the Stefan–Boltzmann law [Ste79, Bol84]:

(17)

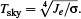




Consequently, we can incorporate solar irradiation data as a Dirichlet boundary condition specifying the effective temperatures on the sky‐dome geometry **T**_*D*._ As described in our supplement, we split the temperature vector **T** into unknown and known parts, **T** = [(**T**_*U*_)^T^, (**T**_*D*_)^T^]^T^. and solve Eq. (14) for the unknown temperature variables:

(18)

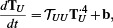




where *𝒯_UU_* describes interactions among the unknown degrees of freedom, and the right‐hand side vector due to Dirichlet boundaries is 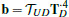.

In summary, we apply a spatial FEM discretization to the radiative heat transport problem, §4.1, which allows us to pre‐compute the transport operator via *photon tracing*, Eq. (16), and solve a system of ordinary differential equations in time, Eq. (18). In the following section, we describe how to solve both steady‐state, as well as transient radiative heat transport problems by either forcing the left‐hand side of Eq. (18) to vanish, or applying a common implicit Euler time discretization scheme. In either case, the fourth‐order term *T*(*x*)^4^ does not cause non‐linear interactions between degrees of freedom due to our spatial discretization. Recall that **T** ^4^ simply contains point‐wise fourth powers (*T_i_* ^4^). Consequently, we solve the remaining non‐linear system of equations using Newton's method, which converges robustly and quickly.

## 5. Implementation details

In practice, the first step is to load the mesh data, either from a *glTF* [Khr22] file, with the additional physical material attributes defined per object with custom attributes or, via a custom‐made plugin directly from the geometry in the computer‐aided design (CAD) application *Rhino3D* (see Section 5.3), using *Attribute User Text* to specify material properties. In order to prevent discontinuities on surfaces, vertices and normals should be shared between triangles. We store the radiative transport operator *𝒯*, Eq. (14), in an *N × N* matrix.

### 5.1. Photon tracing

*Photon tracing* is performed on the GPU using RTX raytracing in Vulkan. In the ray‐tracing shader, each GPU thread is assigned one triangle for which it traces a batch of *M* photons. All photons carry unit weight, which is added using atomic operations to the matrix entries of the corresponding vertices when absorbed. This procedure results in a matrix that holds the number of photons that were emitted around vertex *i* and absorbed at vertex *j* in each entry *i j*. Finally, we normalize each column by the corresponding total amount of emitted photons around each vertex, which gives us the ratio of absorbed to total amount of emitted photons. By simply counting photons first and scaling the matrix later, we save a large amount of repeated calculations, which results in more efficient shader code, and avoid the need for floating‐point atomics.

For our prototype implementation, we used a simplified material model using diffuse and specular reflection coefficients *r_d_* and *r_s_*. To perform *Russian Roulette*, we randomly select the type of reflection or absorption according to the reflection coefficients (diffuse *rd*, specular *r_s_)* and a random variable ξ, uniform in [0,1], as follows [Jen01]:


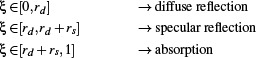




In case of diffuse reflection, we continue the photon's path in a new direction, again drawn cos‐weighted from the hemisphere around the surface normal. For specular reflections, we instead use the direction of ideal reflection. More sophisticated BRDF models can be used as well [Jen96]. In our implementation, we also do not account for wavelength‐dependent effects and instead simulate the total radiation over the entire spectrum. This is sufficient in the early phase of architectural design, since details of the used materials are not exactly specified yet. However, our approach could be extended to account for wavelength‐dependent effects if necessary.

Environment irradiation, e.g., from a discrete sky dome representation (see Section 4.3), is usually defined using radiometric units. For use in our thermal framework, the sky radiosity values are converted to the corresponding black body temperatures, as defined by Eq. (17), and incorporated as Dirichlet boundary conditions, see Eq. (18) as well as our supplementary material.

### 5.2. Thermal Simulation

So far, we have derived a spatially discretized model of radiative heat transport, including solar irradiation data as boundary conditions, and described how modern GPU hardware can accelerate the construction of the radiative transport operator, including indirect reflections. The final step to obtain a thermal simulation from this model is to resolve the time derivative in Eq. (18). We implement both a transient (time‐dependent) simulation via backward Euler time stepping, as well as a steady‐state (time‐independent) solution. Applying the backward Euler method (also known as BDF1, derived from a first‐order Taylor expansion of d**T**_*U*_/d*t*) with time step Δ_*t*_ = *t*_*i*+1_ – *t_i_* results in:

(19)

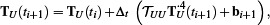




Consequently, we solve the following non‐linear system:


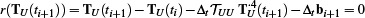




using *Newton's method* in each time step, stabilized with a backtracking line search enforcing decreasing residuals. For the inner linear solver, we use *BiCGSTAB* from the *Eigen* [GJ*10] linear algebra library, where the linearized system matrix is simply 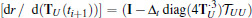. These parts of the computation are done on the CPU.

For the steady‐state solution, we want to find a temperature distribution that does not incur any heat flow over time, i.e. we want to solve for **T**_*U*_ such that d**T**_*U*_/d*t* = 0. Consequently, in the time‐independent case, the residual simplifies to


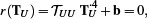




and we apply the same solution strategy as in the transient case.

### 5.3. Pre and post‐processing

We implemented a *C#* plugin for *Grasshopper*, a visual programming language and environment within *Rhino3D*, to export geometry and material specifications from *Rhino3D* to our thermal simulation framework, which then reports simulation results back to the software for interactive visualization. Our *Grasshopper* plugin node currently only supports triangle meshes, so all *Rhino3D* geometry is converted in *Grasshopper* using *TriRemesh* and *Unify Mesh* nodes before it is passed to our simulation node. Our method robustly handles arbitrary geometry; we nevertheless recommend avoiding heavily self‐intersecting meshes or very small triangles. Small elements (relative to neighbors) might receive too few samples on a small area during stochastic photon‐tracing, causing visual artifacts. Overall, uniformly refining the mesh also requires a similarly increased sampling density during photon tracing. However, because we do not build discrete differential operators on the mesh, these issues concern only a few isolated triangles and do not cause the entire solution to numerically degenerate.

Geolocation‐dependent incident solar radiation data is incorporated using sky domes generated using the *Ladybug Cumulative Sky Matrix* and *Ladybug Sky Dome* nodes from the *Ladybug Tools* plugin for *Grasshopper*, which uses *Radiance GENDAYMTX* internally. The sky dome mesh and values are passed into our simulation node and used to construct the internal representation as detailed in Section 4.3.

## 6. Results

In this section, we compare our solution to the well‐known and open‐source finite element solver *Elmer FEM* [MR13, CSC23] and show results computed via *Rhino3D* [Rhi] and our *Grasshopper* [Gra] plugin.

### 6.1. Verification using Elmer FEM

**Figure 5:**
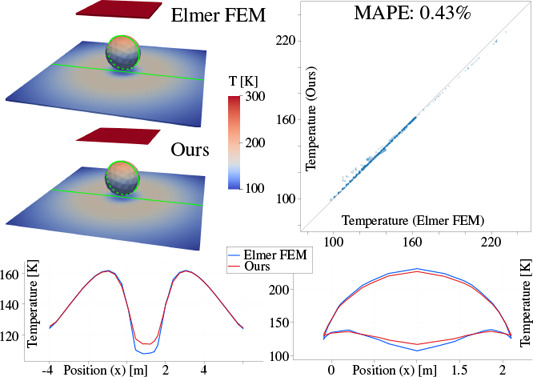
Comparing our solution to *Elmer FEM* on a *simple test* scene. Top left: steady‐state temperature distribution computed by *Elmer FEM*, middle left: our result. Green lines in the 3D views indicate the slice corresponding to the temperature graphs at the bottom (left: floor, right: sphere, *Elmer FEM*: blue, and our approach: red). The scatter plot on the right correlates the temperatures for each surface vertex from both simulations. The mean absolute percentage error (MAPE) of our result is 0.43% relative to *Elmer FEM*, which is visible by the offset of the points from the diagonal. The most noticeable difference is that Elmer's solution remains cooler directly underneath the sphere.

**Figure 6:**
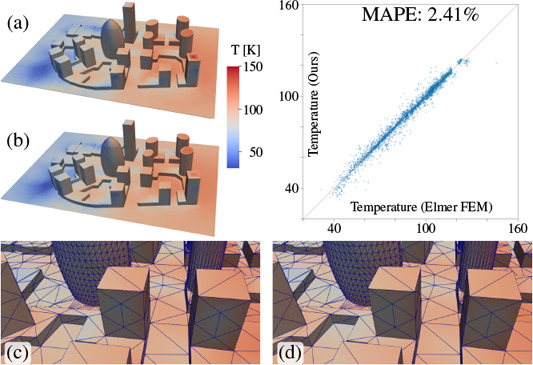
A more complex *city test* scene for comparing our solution to *Elmer FEM*. Analogous to Fig. 5: (a) steady‐state temperature distribution computed by *Elmer FEM*, and (b) our result. The two bottom images show a closeup of the identical surface triangulation used for *Elmer FEM* (c) and our approach (d). For this scene, the mean absolute percentage error (MAPE) is 2.41%, and the plot shows the temperatures for both simulations while the error is indicated by the offset of points from the diagonal.

In order to verify our approach against a state‐of‐the‐art solver, we created two simple test scenes and compared our simulation results to those computed via *Elmer FEM*, an established open‐source multi‐physics finite‐element solver. *Elmer FEM* supports diffuse grey radiation based on view factors, and, in contrast to common tools used in architecture, such as *Ladybug Tools*, allows the simulation of temperature distributions on meshes, similar to our approach. The simulation was set to use a single ray per surface‐patch pair to include shadowing in the view factors, and the same solvers as our implementation, i.e., *Newton's method* and *BiCGSTAB* for its inner linear solver. As Elmer FEM, using standard pair‐wise form factors, only supports diffuse radiation, we restrict our test scenes to ideally diffuse materials for this comparison. We construct the meshes used in the comparison such that both the volumetric *Elmer FEM* mesh and our surface‐only mesh contain the exact same *surface* geometry (identical vertices and indices for surface triangles, see Figure 6, bottom). We then extend this surface geometry to volumetric elements for *Elmer FEM* by offsetting along the inward normals according to the thickness associated with each element. Thus, we maintain a direct correspondence of surface data for comparison. Consequently, each dot in the scatter plots (Figures 5 & 6) denotes an individual vertex. In both test cases, we compute the steady‐state solution.

The first scene (Fig. 5) consists of a small plate with a constant temperature of 300° K placed above a sphere and a larger plate with an initial temperature of zero Kelvin. For this *simple test* scene, the view factor computations in *Elmer FEM* took 13.2 seconds, and the non‐linear solver took 56.2 seconds for 17 iterations to compute the steady‐state temperature distribution. Our implementation took 2.87 seconds to compute the transport operator and 0.038 seconds to calculate the steady‐state solution (see also Table 1), which is roughly a 23× speedup. As the non‐linear solvers of both our and Elmer's simulation run on the CPU, the speed‐up is mostly due to our simplified handling of the fourth‐order term via piecewise‐constant interpolation, compared to Elmer's standard linear finite elements. For the view‐factor computation, our hardware‐accelerated approach traces substantially more rays, but still requires significantly shorter runtime.

**Table 1:** Overview of the mesh size, number of traced photons (top), and corresponding timings (bottom) for each processing step in the simple test (Fig. 5), city test (Fig. 6), hot spot (Fig. 7), and transient city (Fig. 8) scenes respectively.

	Example	Fig. 5	Fig. 6	Fig. 7	Fig. 8
Count	Vertices	1067	2956	6382	13512
	Triangles	1986	5844	7670	18392
	Photons	50 k	50 k	200 k	200 k
Seconds	Transport Op.	2.87	3.35	4.3	30.6
	Steady State	0.038	0.526	9.56	13.9
	Time Step	0.006	0.049	0.479	1.61

**Figure 7:**
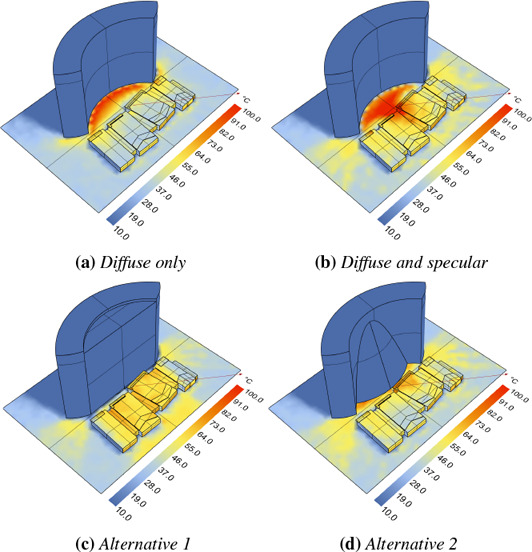
Comparison of diffuse‐only (a) to diffuse and specular transport (b) using our approach. Support for specular transport enables detection of problems such as heat concentration due to a highly reflective and concave building facade. Possible solutions include making the facade diffuse (a), or adjusting its shape as shown in the two examples (c) and (d).

The second scene, shown in Figure 6, includes a more complex block of buildings, initially at zero Kelvin, which receives heat from a plate, at constant 300° K, suspended above. For this more complex *city test* scene, the view factor computations in *Elmer FEM* took 97.7 seconds, and the solver took 14.8 minutes for 34 iterations to compute the steady‐state temperature distribution. Our implementation took 3.35 seconds to compute the transport operator and 0.526 seconds to calculate the steady‐state solution (see Table 1), which is roughly a 250× speedup. These results show that our approach can compute the steady‐state solution for both test scenes much faster. Note that transient simulations using a backward Euler scheme effectively lead to a very similar (but numerically regularized) system that must be solved in each time step. While the cost of view‐factor computation amortizes over the course of a transient simulation, the speed‐up of the non‐linear solver persists as in the steady‐state case.

Comparing our solution with *Elmer FEM* as the reference, our results are well matched, with a mean absolute percentage error (MAPE) of 0.49% for the simple test (Fig. 5) and 2.41% for the more complex scene including multiple buildings (Fig. 6). The differences stem mostly from the residual error inherent to our stochastic *photon tracing approach* and the difference in finite element discretization: volume (*Elmer FEM*) vs. shell (ours).

### 6.2. Examples using Rhino and Grasshopper

In the context of the early‐phase workflow of architectural design, computational support should be fully integrated into well‐known design tools to be useful. Therefore, we demonstrate the application of our approach using the corresponding *Grasshopper* plugin in *Rhino3D* on the example of two use cases, i.e., iterative design and identification of hot spots and transient thermal analysis over several days.

In the design of buildings or larger complexes, it is important to spot possible design flaws or problems early on, since later changes incur higher costs. For example, concave and highly glossy or nondiffuse reflective facades have the potential to focus the sun's energy onto small areas in which the thermal load increases significantly. Common thermal simulation tools, such as *Elmer FEM*, often only account for diffuse radiative transport, which cannot reveal such concentrations of radiation due to non‐diffuse transport.

In contrast, our approach also supports non‐diffuse radiation. Figure 7 shows an example comparing diffuse‐only radiative transport to specular (i.e., non‐diffuse) transport. It illustrates how a highly specular reflective and concave building can cause an unwanted high concentration of heat radiation in a small area (Fig. 7b). To mitigate such a hotspot without changing the design or shape of the facade, a designer would need to remove any material contributing to the specular reflections, resulting in a diffuse‐only scenario (Fig. 7a). If a highly specular facade is desired, and the facade design is free to change, the designer can iterate through different designs and quickly test if there are still hotspots present, as illustrated by the two examples in Fig. 7c and 7d.

**Figure 8:**
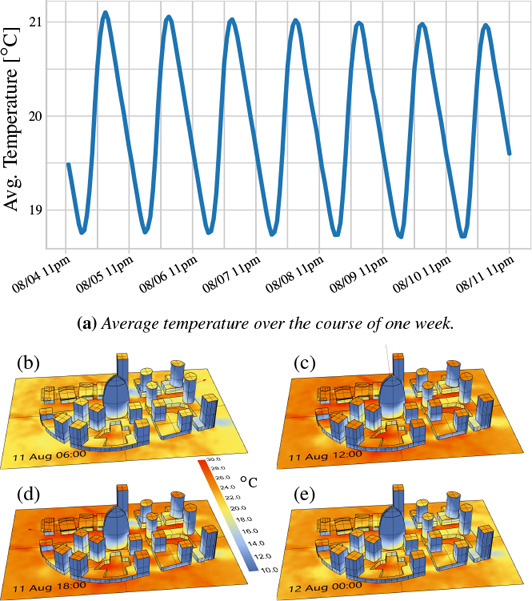
Average temperature plot (a) over a week, and images (b‐e) showing the corresponding temperature distribution for selected hours over a day.

Another aspect in the early design phase of architectural projects is the thermal analysis over time, i.e., change of heat distributions over several hours or days. In Figure 8 we illustrate how our approach is used to simulate the surface temperature change due to thermal radiation in a model of several city blocks. The plot (Fig. 8a) shows the average surface temperature over the course of seven days, while the images below show the heat distribution on the surfaces for a selection of four different hours.

### 6.3. Real World Example

**Figure 9:**
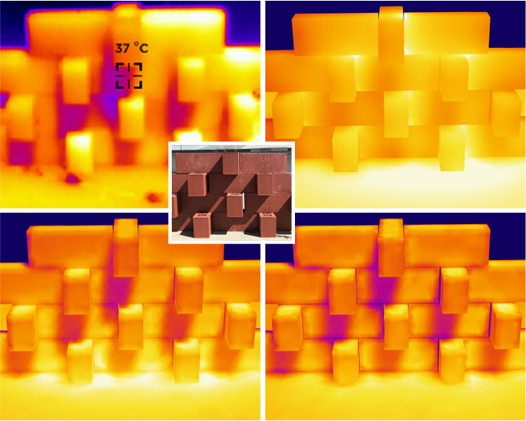
Qualitative comparison to a real‐world example: a wall from the *Climate Active Bricks* project [Mol23] as seen from a thermal (top left) and a regular (central inset) camera; real‐world images courtesy of P. Molter. We recreate this scenario using a parallel emitting surface (bottom left), as well as a diffuse emitter (bottom right). We also compare the latter result to a simulation using *Elmer FEM* (top right). All three simulations are visualized with the same color scale, which mimics the output of the thermal camera.

For a qualitative comparison to a real‐world scenario, we approximate the geometry of a brick wall published by the *Climate Active Bricks* project [FMCD22, FDMC23]. They developed a self‐shaded building facade, i.e., a special type of brick wall, shown in Figure 9 (top). We compare our simulated result (Fig. 9 bottom, right) with their thermal‐imaging photograph of the real wall (bottom, left). This example illustrates how our approach could also be used to support real‐world architectural research and development on the scale of facades instead of the urban scale. Please note that this experiment is preliminary and limited to a qualitative comparison, as we do not simulate the sensitivity of the thermal camera, nor do we have access to the exact material or illumination parameters of the original experiment. Nevertheless, from the point of view of the early design phase, our results qualitatively match the observed behavior well, which is sufficient to guide designers toward promising solutions. We also compare to *Elmer FEM* for this scenario, using a diffuse instead of a parallel emitter. Due to memory limitations, Elmer takes about 1.5 hours in this case; our result (using 1*M* parallel rays) takes about 22 s for photon tracing and 2 s to find the steady‐state solution. Note that Elmer's view‐factor‐based approach struggles with the narrow gaps between the bricks, which unrealistically heat up. Due to numerical issues, we had to include heat conduction (which acts as a smoothing regularizer) in the Elmer simulation, resulting in a noticeably smoother overall result compared to our simulation. In the future, we plan to also include conduction in our framework.

### 6.4. Geometry Sizes and Timings

Table 1 shows the geometry sizes and timings for each of our shown experiments. The timings are given for single invocations of the different processing steps. For the *transient city blocks* experiment, the total processing time depends on the number of simulated hours and is, therefore, a multiple of the reported (single invocation) time step, plus a single processing step for the pre‐computed transport operator. For the other steady‐state experiments, the total processing time is the sum of the steady‐state and transport operator computation timings. Additionally, in Table 2, we analyze the performance of our approach with respect to the number of finite elements (scene triangulation resolution) and the number of photons per triangle used for the computation of the transport operator via *photon tracing*.

**Table 2:** Timings (execution time in seconds) and mean absolute percentage error (MAPE) for different photons counts (rows) and mesh resolutions (columns) of the city blocks model (shown in Figure 8). “Transport” refers to the time taken for precomputing the transport operator on the GPU; “Time Step” denotes the average time required to compute one time step representing one hour of simulated time; “Steady St.” gives the timing for solving the nonlinear steady state problem. The reference temperatures for the MAPE calculations are the steady‐state temperatures based on the transport matrix calculated using the maximum ray count (1000k). Since there is no one‐to‐one correspondence between the vertices of different mesh resolutions (columns), the MAPE are only directly comparable within the rows of a column, and not across.

Photons	Num.	Vertices	22551	15265	10254	5044
		Triangles	33924	20882	12387	3986
1000k	Sec.	Transport	75.30	36.20	16.400	6.660
		Time Step	4.380	2.160	1.120	0.366
		Steady St.	36.90	23.80	5.200	3.330
	T	MAPE	0.0 (per column reference)			
100k	Sec.	Transport	7.540	3.610	1.650	0.685
		Time Step	4.540	2.270	1.150	0.356
		Steady St.	46.00	26.80	10.70	3.500
	T	MAPE	0.839	0.844	0.737	0.724
10k	Sec.	Transport	0.753	0.362	0.165	0.077
		Time Step	6.150	3.050	1.140	0.517
		Steady St.	51.30	28.60	12.10	3.700
	T	MAPE	1.841	1.682	1.467	1.731
1k	Sec.	Transport	0.094	0.038	0.019	0.010
		Time Step	4.390	2.240	1.130	0.358
		Steady St.	61.90	28.50	11.40	5.510
	T	MAPE	5.218	4.595	3.783	4.240

For vertex counts ranging from 5*k* to 20*k* and photon counts ranging from 1*k* to 1000*k*, the pre‐computation of the transport operator takes 0.01 to 75.3 seconds. The computation of the steady state takes from 3.3 to 61.9 seconds depending on the number of vertices. Interestingly, the higher the photon count, the faster the solver can compute the steady state, presumably due to the higher accuracy of the transport operator. To analyze the MAPE of the computed temperature distributions, we compared against the results computed using the transport operator with most accuracy, i.e., calculated using 1000*k* photons. Depending on the photon count, the MAPE ranges from 0.72 to 5.21 percent. Note that the MAPE values are only comparable within each column, across different photon counts. They are not directly comparable across columns since we did not compare temperature distributions across geometries with different vertex counts. All results were computed on a system with an *AMD Ryzen 5 1600X* CPU, 16 GB of RAM and a *NVIDIA GeForce RTX 3070* GPU with 8 GB of VRAM. The analysis indicates how our approach can facilitate rapid design iterations. It is possible to quickly produce results by running the computations at a lower mesh resolution and with fewer photons until a few final design candidates are developed, and then analyze their performance in more detail at higher mesh resolutions and photon counts.

## 7. Discussion and Limitations

In this section, we discuss different properties, characteristics, and limitations of our approach and prototype implementation, as well as future work. Computing and caching a global transport operator makes our approach very efficient for the simulation of transient radiative heat exchange as long as the geometry does not change. Because we store the full, global transport matrix, which is in general dense, memory consumption scales quadratically in the number of vertices, limiting the size of scenes that can be efficiently processed. Furthermore, any change of scene geometry currently requires a re‐computation of the transport operator. To alleviate these constraints, some form of hierarchical, adaptive, or iterative schemes inspired by existing optimizations for radiosity may be applied in future work. An ideal approach would need to preserve the efficiency of a cached global transport operator while at the same time lowering the memory requirements and supporting effective updates due to changes in geometry. Addressing all of these requirements in one unified approach poses a significant challenge. One insight is that the transport matrix already holds information on which part of the geometry is affected by another part, possibly enabling efficient update heuristics. This information can also be used to visualize and analyze the origin or distribution of energy between different parts of the scene.

In our current implementation, we compute the transport operator on the GPU, and use a solver running on the CPU for the heat equation. Accelerating all computations via the GPU, including the heat equation solver, may speed up computation even more, especially for bigger scenes, and would also eliminate some of the overhead of transferring data between CPU and GPU memory. Furthermore, we see potential in using solvers that deal with partially computed or incrementally updated transport matrices, in order to lower memory requirements or support geometry updates.

In our experiments, we simulate temperatures on the basis of the total energy transported across the entire electromagnetic spectrum, instead of considering multiple spectral bands for, e.g., near and far infrared. Furthermore, we assumed all material properties to be independent of wavelength, which is not the case in general. However, those are not limitations of our general approach, which is able to incorporate these aspects at the expense of additional computational overhead.

For the concrete experiments shown in this paper, we select plausible simulation and material parameters, but rely on actual measurements only for the sky model. So, although we verified our results using *Elmer FEM*, the reported temperatures are not currently verified against real‐world data. Such a predictive analysis would require careful calibration and real‐world measurements for all scene parameters, as well as an informed design of the experiment. For our experiments, we clamp the effective sky temperature to a selected minimum value of 63.15 Kelvin, since at night the sky model reports zero radiation, which would cause an implausibly strong overnight cooling effect.

In our approach, we focused on heat exchange via radiation. Since we already use a finite element approach, it can be extended to thermal conduction using the same framework as well. The extension to, and integration of, all three modes of heat exchange, including convection, while maintaining a workflow consisting of fast design iterations, would be a significant step forward towards a unified framework for thermal simulation, and we consider this challenge as future work.

## 8. Conclusion

We have presented an approach for efficiently simulating heat exchange via radiation, inspired by physically‐based rendering techniques, which we extended and adapted for the thermal domain. Our approach can support rapid iteration and design evaluation as common in the early phase of urban and architectural planning. It supports arbitrary geometry and radiation transport, i.e., diffuse as well as non‐diffuse or glossy reflections. It combines a carefully chosen finite element surface discretization with a photon tracing approach that utilizes the latest advancements in hardware‐accelerated ray tracing on the GPU. By pre‐computing and caching the global transport operator we can efficiently compute transient solutions for the temperature distribution on all surfaces of a scene. We verified our approach using *Elmer FEM*, an established finite element solver, and illustrated the use of our *Rhino3D* and *Grasshopper* integration (source code: Grasshopper plugin, standalone program) on examples in the context of early‐phase architectural design of building complexes. We see our approach as an important step towards a more efficient and unified simulation of thermal transport in complex scenes.

## Acknowledgments

This work has received funding from the Austrian Science Fund (FWF) project F 77 (SFB “Advanced Computational Design”), the Vienna Science and Technology Fund (WWTF) project ICT22‐028 (“Toward Optimal Path Guiding for Photorealistic Rendering”), as well as from King Abdullah University of Science and Technology (KAUST) baseline funding (BAS/1/1679‐01‐01) and the KAUST/NEOM Green Building Initiative (project ID ORS 4554). We also thank Mostapha Sadeghipour Roudsari for giving us permission to use the city model shown in Fig. 1, 4, 6, and 8, as well as Philipp Molter for providing the real‐world images in Fig. 9.

## Supporting information

Supporting Information
