# Supplementary material for: Precomputed Radiative Heat Transport for Efficient Thermal Simulation
Source: Comput Graph Forum. 2023 Nov 5;42(7):e14957. doi: 10.1111/cgf.14957 (PMC10946482; doi:10.1111/cgf.14957)
Supplement: Supplementary file 1 — Supporting Information [file CGF-42-0-s001.pdf]

Supplementary material

S.1. FEM discretization

Here, we provide a short summary on the derivation of Eq. (10) following the finite element approach. Starting from Eq. (8), we first multiply by a test function (for which we choose any interpolation basis function I_j according to Galerkin's method):

$$\frac{\mathrm{d}T}{\mathrm{d}t} I_j(x) = \frac{\varepsilon(x)}{c_p(x)\rho(x)h(x)} \left(E(x) - \sigma T(x)^4\right) I_j(x).$$

Next, we integrate over the entire domain, and substitute the piecewise-constant approximation for the temperature field. We also assume that the material properties are similarly given as piecewise-constant data, consequently:

$$\int_\Omega \frac{\mathrm{d}\left(\sum_i I_i(x)T_i\right)}{\mathrm{d}t} I_j(x)\ \mathrm{d}x = \int_\Omega \frac{\varepsilon_j}{c_j\rho_j h_j} \left(E(x) - \sigma\left(\sum_i I_i(x)T_i\right)^4\right) I_j(x)\ \mathrm{d}x.$$

On the left-hand side, clearly only the temperature coefficients T_i are time-dependent, while only the indicator functions vary spatially. We can therefore re-arrange terms and exchange the order of integration, summation, and differentiation. Noting that the product $I_i I_j$ is 1 when $i = j$ and 0 otherwise, only one term of the sum remains and the left-hand side simplifies as follows:

$$\int_\Omega \frac{\mathrm{d}\left(\sum_i I_i(x)T_i\right)}{\mathrm{d}t} I_j(x)\ \mathrm{d}x = \sum_i \frac{\mathrm{d}T_i}{\mathrm{d}t} \int_\Omega I_i(x)I_j(x)\ \mathrm{d}x = \frac{\mathrm{d}T_j}{\mathrm{d}t} A_j.$$

Similarly, the fourth-order term on the right-hand side simplifies because at any point x only one term of the sum is non-zero (also note $I_i^4 = I_i$ as the indicator is either 0 or 1):

$$\left(\sum_i I_i(x)T_i\right)^4 I_j(x) = T_j^4 I_j(x).$$

Note that, choosing piecewise-constant interpolation and test functions here conveniently simplifies the fourth power of the sum. Had we used more common piecewise-linear functions instead, the sum would consist of three terms per triangle, and the fourth-power would generate a total of 15 terms (including 12 additional mixed terms, where temperature variables at the corners of the triangle interact in its interior due to linear interpolation). Furthermore, with linear functions $I_i I_j$ would also be non-zero for any pair of adjacent nodes i and j.

Combining the aforementioned simplifications, index i no longer occurs. For consistency of notation, we replace j with i in the following, so we now have

$$\frac{\mathrm{d}T_i}{\mathrm{d}t} A_i = \int_\Omega \frac{\varepsilon_i}{c_i\rho_i h_i} \left(E(x)I_i(x) - \sigma T_i^4 I_i(x)\right) \mathrm{d}x.$$

We then split the integral on the right-hand side and rearrange constants to obtain

$$\frac{\mathrm{d}T_i}{\mathrm{d}t} A_i = \frac{\varepsilon_i}{c_i\rho_i h_i} \left(\int_\Omega E(x)I_i(x)\ \mathrm{d}x - \sigma T_i^4 A_i\right),$$

where we again use $A_i = \int_\Omega I_i(x)\,\mathrm{d}x$ on the right-most term. Finally, we divide by A_i and also note that the remaining integral results in the incident flux on the support region of I_i, which we denote as $\check{\Phi}_i = \int_\Omega E(x)I_i(x)\ \mathrm{d}x$, to arrive at Eq. (10):

$$\frac{\mathrm{d}T_i}{\mathrm{d}t} = \frac{\varepsilon_i}{c_i\rho_i h_i} \left(\frac{\check{\Phi}_i}{A_i} - \sigma T_i^4\right).$$

S.2. Boundary conditions

Here, we briefly outline how to incorporate the Dirichlet boundary conditions, describing external (solar) irradiation, into the temperature simulation. As discussed in the main paper, Eq. (10) (restated above) can be concisely expressed in matrix-vector notation as Eq. (14), i.e., $\mathrm{d}\mathbf{T}/\mathrm{d}t = \mathcal{T}\,\mathbf{T}^{\cdot 4}$. Recall that $\mathbf{T}$ is a vector of all (per-vertex) temperature variables, and superscript "·4" refers to taking the component-wise fourth power of this vector. We now outline the derivation of Eq. (18) from this starting point. Our boundary data specifies effective temperature values at the vertices of the emitter geometry. Therefore, some entries in the temperature vector $\mathbf{T}$ become known due to boundary conditions ($\mathbf{T}_D$), while most entries remain unknown variables ($\mathbf{T}_U$). Theoretically, we can now sort $\mathbf{T}$ such that all unknown components are listed before known data, i.e., we assume without loss of generality $\mathbf{T} = [\mathbf{T}_U^\mathsf{T}\ \mathbf{T}_D^\mathsf{T}]^\mathsf{T}$. Applying the same sorting and partitioning to the transport operator $\mathcal{T}$ expands the temperature equation to

$$\frac{\mathrm{d}}{\mathrm{d}t}\begin{bmatrix} \mathbf{T}_U \\ \mathbf{T}_D \end{bmatrix} = \begin{bmatrix} \mathcal{T}_{UU} & \mathcal{T}_{UD} \\ \mathcal{T}_{DU} & \mathcal{T}_{DD} \end{bmatrix} \begin{bmatrix} \mathbf{T}_U^{\cdot 4} \\ \mathbf{T}_D^{\cdot 4} \end{bmatrix}.$$

As the given boundary data, $\mathbf{T}_D$, is known throughout the simulation, we can discard the second row of this block-vector equation. Retaining only the first row reads

$$\frac{\mathrm{d}\mathbf{T}_U}{\mathrm{d}t} = \mathcal{T}_{UU}\mathbf{T}_U^{\cdot 4} + \mathcal{T}_{UD}\mathbf{T}_D^{\cdot 4}.$$

Denoting the last term as $\mathbf{b} = \mathcal{T}_{UD}\mathbf{T}_D^{\cdot 4}$ results in Eq. (18).

S.3. Material Parameters

Table S1 summarizes material parameters for our various numerical experiments.

© 2023 The Authors.
Computer Graphics Forum published by Eurographics and John Wiley & Sons Ltd.

Table S1: *Material properties and simulation parameters used for the simulations shown in the various Figures. Emissivity* ε*, diffuse reflectivity* r_d*, specular reflectivity* r_s*, mass density* ρ *[kg / m*^{3}*], specific heat capacity* c_p *[J / (kg K)], shell thickness h [m], and fixed boundary temperature T [K] for emitting objects.*

Figure	Scene element	ε	r_d	r_s	ρ	c_p	h	fixed T
5, 6	Mesh	0.5	0.5	0.0	1000	1000	0.1	-
	Radiator	0.5	0.5	0.0	1000	1000	0.1	300
7	Ground	0.7	0.3	0.0	2000	1000	10	-
	Small Building	0.5	0.5	0.0	2000	1000	0.5	-
	Main Building (a)	0.0	1.0	0.0	2000	1000	0.5	-
	Main Building (b, c, d)	0.0	0.0	1.0	2000	1000	0.5	-
8	City	0.5	0.5	0.0	2000	1000	0.5	-
9	Mesh	0.8	0.2	0.0	1800	800	0.013	-
	Radiator (diffuse)	0.8	0.2	0.0	1800	800	0.013	930
	Radiator (parallel)	0.8	0.2	0.0	1800	800	0.013	345

© 2023 The Authors.
Computer Graphics Forum published by Eurographics and John Wiley & Sons Ltd.
